# Two complete chloroplast genomes of an endangered orchid species, *Pelatantheria scolopendrifolia* (Orchidaceae), in Korea

**DOI:** 10.1080/23802359.2018.1437815

**Published:** 2018-02-12

**Authors:** Seon A. Yun, Hyun-Deok Son, Hyoung-Tak Im, Seung-Chul Kim

**Affiliations:** a Department of Biological Sciences, Sungkyunkwan University, Suwon, Republic of Korea;; b Mokpo Natural History Museum, Mokpo, Republic of Korea;; c Department of Natural Sciences, Chonnam National University, Gwangju, Republic of Korea

**Keywords:** Chloroplast genome, *Pelatantheria scolopendrifolia*, endangered species, *ndh* gene, Korean peninsula

## Abstract

We determined the complete chloroplast genome sequence of two individuals of *Pelatantheria scolopendrifolia*, an endangered orchid species in Korea. The total chloroplast genome size of Mokpo (MG752972) and Naju (MG752973) population was 146,971 bp and 146,848 bp, respectively. The chloroplast genome contained 106 genes, including 72 protein-coding genes, 30 tRNA genes, and four rRNA genes. Variation in gene contents and structures was not found between two individuals. We found truncated or deleted *ndh* genes in *P. scolopendrifolia.* Phylogenetic analysis, based on the whole chloroplast genome sequences of 25 species of Orchidaceae, showed that *P. scolopendrifolia* was most closely related to *Gastrochilus fuscopunctatus*.

*Pelatantheria scolopendrifolia* (Makino) Aver. is a creeping evergreen epiphytic or lithophytic orchid and has small flowers (ca. 6–7 mm diameter in full blooming), leathery leaves (6–10 mm long), and many noded and branched slender stems. It occurs in East Asia, i.e. Korea, China (Tsi et al. 1999), and Japan (Ohasi et al. 2015), and with the exception of one inland population (Naju population), *P. scolopendrifolia* is highly restricted to southwestern coastal areas and small islands of Jellanamdo province and Jejudo Island in Korea (Lee 2011). Less than 10 populations are known with yearly variable population sizes. The extent of occurrence of this species was estimated to be 2000 km^2^ (National Institute of Biological Resources 2014). Given illegal collection for ornamental use and trading, habitat disturbance, and very small population sizes, *P. scolopendrifolia* is recognized as EN B2b(iii,iv)c(iii,iv,v) in the Korean Red List (National Institute of Biological Resources 2014). Despite their value and importance for conservation, very little is known for breeding system, genetic diversity, and population genetic structure of 10 known populations in Korea. In this study, we sequenced the complete chloroplast genome of two wild individuals collected from two geographical regions with different habitats, one inland population (Naju; voucher specimen CNU173926) and the other coastal population (Mokpo; voucher specimen CNU173074), in Korea. Total DNA was isolated from fresh leaves using GeneAll Exgene Plant SV mini kit (Geneall Biotechnology Co., Seoul, Korea) according to the instructions of the manufacturer. The genomic sequencing by an Illumina Miseq (Illumina Inc., San Diego, CA) platform was performed and short reads were assembled using SPAdes 3.6.1 (Bankevich et al. 2012) and Velvet (Zerbino and Birney 2008). Annotations were performed using BLAST of the National Center for Biotechnology Information and tRNAscan-SE with default setting (Lowe and Chan 2016). The complete chloroplast genome sequences of *P. scolopendrifolia* were submitted to the GenBank, and their accession numbers were acquired; coastal Mokpo population (MG752972) and inland Naju population (MG752973).

The complete chloroplast genome of coastal (MG752972) and inland population (MG752973) was 146,971 bp (LSC, 86,096 bp; SSC, 11,735 bp; IRa and IRb, 24,570 bp) and 146,848 bp (LSC, 85,984 bp; SSC, 11,730 bp; IRa and IRb, 24,567 bp), respectively. The chloroplast genome contained 106 genes, including 72 protein-coding genes, 30 tRNA genes, and four rRNA genes. We found mutations and/or deletions in 11 protein-coding genes (*atp*A, *rpo*C1, *rbc*L, *rps*18, *clp*P, *rpo*A, *inf*A, *rpl*16, *ndh*E, *ycf*1, and *ycf*2) and two tRNA genes (*trn*K-UUU and *trn*L-UAA) between two individuals. Of 11 *ndh* genes in plant chloroplast genomes, four (*ndh*B, C, E, and J) were truncated, while the remaining seven genes were lost (most likely transferred to the mitochondrial genome; Lin et al. 2015). For the phylogenetic analysis, 25 complete chloroplast genome sequences of Orchidaceae were obtained and aligned using MAFFT v7.222 (Katoh and Standley 2013). Maximum likelihood (ML) analysis was performed using IQ-TREE v.1.4.2 (Nguyen et al. 2015) with 1000 bootstrap replications based on GTR +4R model. The phylogenetic tree showed that *P. scolopendrifolia* was closely related to *Gastrochilus fuscopunctatus* (Figure 1).

**Figure 1.**
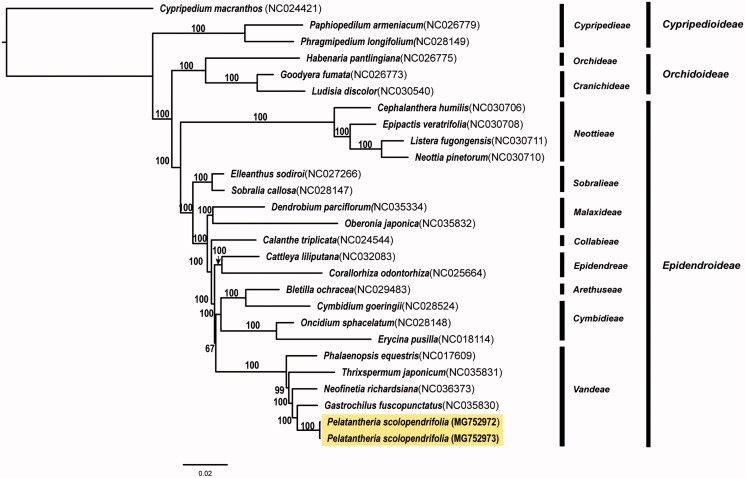
Maximum likelihood tree of *Pelatantheria scolopendrifolia* (two accessions) and 25 representative species in the family Orchidaceae. The bootstrap support values >50% are shown above the branches.
